# The Transcription Factors Atf1 and Pcr1 Are Essential for Transcriptional Induction of the Extracellular Maltase Agl1 in Fission Yeast

**DOI:** 10.1371/journal.pone.0080572

**Published:** 2013-11-05

**Authors:** Hiroaki Kato, Shintaro Kira, Makoto Kawamukai

**Affiliations:** 1 Department of Life Science and Biotechnology, Faculty of Life and Environmental Science, Shimane University, Matsue, Japan; 2 Department of Biochemistry, Shimane University School of Medicine, Izumo, Japan; 3 PRESTO, Japan Science and Technology Agency (JST), Saitama, Japan; Cancer Research UK London Research Institute, United Kingdom

## Abstract

The fission yeast *Schizosaccharomyces pombe* secretes the extracellular maltase Agl1, which hydrolyzes maltose into glucose, thereby utilizing maltose as a carbon source. Whether other maltases contribute to efficient utilization of maltose and how Agl1 expression is regulated in response to switching of carbon sources are unknown. In this study, we show that three other possible maltases and the maltose transporter Sut1 are not required for efficient utilization of maltose. Transcription of *agl1* was induced when the carbon source was changed from glucose to maltose. This was dependent on Atf1 and Pcr1, which are highly conserved transcription factors that regulate stress-responsive genes in various stress conditions. Atf1 and Pcr1 generally bind the TGACGT motif as a heterodimer. The *agl1* gene lacks the exact motif, but has many degenerate TGACGT motifs in its promoter and coding region. When the carbon source was switched from glucose to maltose, Atf1 and Pcr1 associated with the promoters and coding regions of *agl1*, *fbp1*, and *gpx1*, indicating that the Atf1-Pcr1 heteromer binds a variety of regions in its target genes to induce their transcription. In addition, the association of Mediator with these genes was dependent on Atf1 and Pcr1. These data indicate that Atf1 and Pcr1 induce the transcription of *agl1*, which allows efficient utilization of extracellular maltose.

## Introduction

The disaccharide maltose must be hydrolyzed into two glucose molecules for it to be utilized as a carbon source by the cell. The enzyme maltase catalyzes this hydrolysis, thereby allowing efficient utilization of maltose. In the budding yeast *Saccharomyces cerevisiae*, maltose is transported into cells through maltose-specific transporters and is hydrolyzed into glucose by intracellular maltases [1]. By contrast, the fission yeast *Schizosaccharomyces pombe* cannot utilize maltose when the glucose transporter Std1 is inactivated [2], suggesting that maltose hydrolysis occurs outside cells. Instead of maltose being transported into cells, fission yeast secretes the extracellular maltase Agl1 to hydrolyze maltose into glucose, which is then transported into cells [3,4]. Conversely, other studies have suggested that fission yeast uses the plant sucrose transporter-like protein Sut1 to transport maltose into cells [5], where it is hydrolyzed by the maltase-like protein Mal1 [6]. However, the physiological importance of Sut1 and Mal1 in hydrolysis of maltose by fission yeast remains unclear, since they have only been analyzed in other species. In the fission yeast genome, there are two genes that encode uncharacterized proteins that are similar to Agl1 (*SPAC1039.11c* and *SPAC30D11.01c*); however, the involvement of these genes in maltose hydrolysis has not been studied. In addition, although transcriptional regulation of factors that are responsible for maltose utilization in *S. cerevisiae* have been well-studied [1], the factors that regulate *agl1* expression in *S. pombe* have not been identified. 

 The fission yeast basic leucine zipper transcription factors Atf1 and Pcr1 are homologues of mammalian ATF-2 and CREB, respectively. The heterodimer of Atf1 and Pcr1 binds sequences that contain a TGACGT motif *in vitro* [7,8]. This heterodimer participates in various cellular events that occur on chromatin, such as meiotic recombination, induction of stress-responsive and sexual differentiation-associated genes, and maintenance of heterochromatin structure at the mating-type locus in the absence of RNAi activity [8-11]. Several studies have shown that Atf1 and Pcr1 bind to TGACGT motifs in the promoters of Atf1 target genes *in vivo*, even under unstressed conditions, and that their binding patterns are not drastically altered after the transcription of these target genes is induced in response to oxidative and osmotic stresses [12-14]. Atf1 has also been shown to become associated with the promoter of the *fbp1* gene, which is required for gluconeogenesis, in response to glucose depletion [15,16]. Therefore, activity of Atf1 as a transcription factor appears to be regulated also at the level of chromatin binding.

 Atf1 is phosphorylated by Sty1 mitogen-activated protein kinase (MAPK) following exposure of cells to environmental stresses, such as high concentrations of KCl [17]. Atf1 is also phosphorylated by Pmk1 MAPK when cells are treated with micafungin, a drug that induces cell wall damage [18]. However, an Atf1 protein in which all eleven MAPK consensus phosphorylation sites are mutated can still bind the promoters of its target genes and activate their transcription; therefore, phosphorylation of Atf1 by MAPKs does not appear to play an important role in transcriptional activation [19]. Sty1 is recruited to the promoters and coding regions of stress-responsive Atf1 target genes in an Atf1- and Pcr1-dependent manner [12,14]. This implies that Atf1 and Pcr1 mediate recruitment of Sty1 to target genes and ensure that transcription of these genes is induced. Despite the importance of Atf1 and Pcr1 in the recruitment of Sty1 to the coding regions of target genes, there is no evidence that Atf1 and Pcr1 associate with these regions. The multiprotein complex Mediator is believed to transmit regulatory signals from sequence-specific activators to the transcriptional complexes that are assembled at the promoters of target genes, and is required for transcription driven by RNA polymerase II [20-23]. However, whether Atf1 and Pcr1 are required for the recruitment of Mediator to specific target genes is unknown.

 In this study, we show that of the four potential maltases in fission yeast, Agl1 is primarily responsible for the efficient utilization of maltose, and that Atf1 and Pcr1 induce transcription of *agl1* when the carbon source is switched from glucose to maltose. Atf1 and Pcr1 associate with the promoters and coding regions of target genes in response to this carbon source change. Accordingly, Med7, an essential Mediator subunit, is recruited to target genes in an Atf1- and Pcr1-dependent manner, suggesting a role for Mediator in Atf1- and Pcr1-dependent induction of transcription. These data indicate that Atf1 and Pcr1 positively regulate transcription of the secreted maltase Agl1, thereby allowing efficient utilization of extracellular maltose.

## Materials and Methods

### Manipulation of fission yeast strains

The fission yeast strains used in this study are listed in Table 1. All the heterothallic strains used in this study were derived from wild-type L972 (*h* ^-^). Deletion and tagging of chromosomal genes were performed using the two-step PCR method [24], pFA6a-based and pCR2.1-based plasmids [25-27], and gene-specific primers (Table S1). To select antibiotic-resistant colonies, 100 mg/l G418 and 150 mg/l hygromycin B were added to YE (0.5% yeast extract and 3% glucose). Successful gene replacement was confirmed by PCR using locus-specific primers. In some experiments, media containing 3% maltose rather than 3% glucose, or media containing both 3% maltose and 3% glucose, were used. A shortage of glucose in the laboratory forced us to use maltose as a carbon source for tetrad dissection, leading to the finding that spores cannot utilize maltose. To switch the carbon source from glucose to maltose, cells exponentially growing in YE medium at 30°C were washed twice with YEM (0.5% yeast extract and 3% maltose) and resuspended in YEM. Cell density was determined using a particle counter (Sysmex PDA-500). 

**Table 1:** Fission yeast strains used in this study.

Strain	Genotype	Source
L972	*h* ^-^	Laboratory stock
L968	*h^90^*	NBRP Yeast
HKS-050	*h* ^-^, *atf1∆::kanMX*	This study
HKS-051	*h* ^-^, *atf1∆::kanMX*	This study
HKS-024	*h* ^-^, *pcr1∆::kanMX*	This study
HKS-025	*h* ^-^, *pcr1∆::kanMX*	This study
KRS-006	*h* ^-^, *gto1∆::kanMX*	This study
KRS-007	*h* ^-^, *gto2∆::kanMX*	This study
KRS-008	*h* ^-^, *agl1∆::kanMX*	This study
KRS-011	*h* ^-^, *mal1∆::hphMX*	This study
KRS-012	*h* ^-^, *sut1∆::hphMX*	This study
KRS-015	*h* ^-^ *, agl1∆::natMX*, *gto1∆::kanMX*	This study
KRS-016	*h* ^-^ *, agl1∆::kanMX*, *gto2∆::hphMX*	This study
KRS-025	*h* ^-^ *, agl1∆::natMX*, *mal1∆::hphMX, gto1∆::kanMX*	This study
KRS-026	*h* ^-^ *, agl1∆::kanMX*, *gto1∆::kanMX, gto2∆::hphMX*	This study
KRS-053	*h* ^-^ *, agl1∆::natMX*, *mal1∆::hphMX, gto1∆::kanMX*	This study
HKS-097	*h* ^-^, *atf1-5FLAG::kanMX*	This study
HKS-224	*h* ^-^, *atf1-5FLAG::kanMX, pcr1∆::hphMX*	This study
HKS-121	*h* ^-^, *pcr1-5FLAG::kanMX*	This study
HKS-222	*h* ^-^, *pcr1-5FLAG::kanMX, atf1∆::hphMX*	This study
KKS-050	*h* ^-^, *med7-5FLAG::kanMX*	This study
HKS-235	*h* ^-^, *med7-5FLAG::kanMX, atf1∆::hphMX*	This study
HKS-237	*h* ^-^, *med7-5FLAG::kanMX, pcr1∆::hphMX*	This study
TTS-047	*h* ^-^, *atf1-5FLAG::kanMX, sty1∆::hphMX*	This study

### Quantitative RT-PCR

Total RNA was extracted as previously described [28]. Total RNA (1 µg) was treated with 122.5 U DNase I (Invitrogen) in a 100 µl reaction for 1 hour at 37°C. The enzyme was denatured at 90°C for 10 minutes, and then 2.5 µl of the solution was added to each reaction tube. Quantitative RT-PCR (qRT-PCR) was performed using a One Step SYBR PrimeScript RT-PCR kit Ver. 1 (TaKaRa, RR066A) and a Thermal Cycler Dice Real-Time System (TaKaRa, TP-800), according to the manufacturer’s instructions. Primers were designed to amplify the C-terminal regions of target ORFs (Table S2). *act1* was used as an internal control because the level of *act1* mRNA does not change when the carbon source is switched from glucose to maltose. All reactions detecting *act1* were performed in the absence and presence of reverse transcriptase to confirm that the samples were not contaminated with genomic DNA. The relative amount of each transcript was calculated with the 2^-∆∆Ct^ method [29] using the cycle threshold value, which was automatically determined by the real-time PCR system by means of the second derivative maximum method [30]. 

### Chromatin immunoprecipitation

Chromatin immunoprecipitation (ChIP) was performed as previously described [31], with improved sonication and immunoprecipitation steps. Sonication using a Bioruptor (Cosmo Bio, UCD-250) was performed for 240 seconds at level H (250 W) in ice-cold water. Sheared DNA was run on a 2% agarose gel in TAE buffer, stained with ethidium bromide, and verified to be shorter than 1 kb. Anti-FLAG M2 (SIGMA, F1804) and anti-CTD 4H8 (Upstate, 05-623) mouse monoclonal antibodies and a rabbit polyclonal antibody against the C-terminal region of histone H3 (Abcam, ab1791) were used to immunoprecipitate FLAG-tagged proteins, RNA polymerase II, and histone H3, respectively. M-280 anti-mouse (or anti-rabbit) sheep antibody-conjugated magnetic beads (200 µl; DYNAL, 112-02 and 112-04) were washed twice with 1 ml buffer 1 (50 mM HEPES-KOH [pH 7.5], 140 mM NaCl, 1 mM EDTA, 1% Triton X-100, and 0.1% Na-deoxycholate), and incubated for 1 hour at 4°C with the appropriate volume of primary antibodies diluted in buffer 1. The beads were again washed twice with buffer 1 to remove unbound antibodies and then incubated for 1 hour with 500 µl of soluble extracts at 4°C. SYBR Premix Ex Taq (TaKaRa, RR041A) and a Thermal Cycler Dice Real-Time System (TaKaRa, TP-800) were used for quantitative PCR (qPCR). The quality of the primers was evaluated as described in the qRT-PCR section. Primers used for qPCR are listed in Table S3. The enrichment level of each target region was calculated relative to that of *act1* as described in the qRT-PCR section.

### Identification of degenerate Atf1-Pcr1 binding sites

The sequences of previously identified Atf1-Pcr1 binding sites [14] were processed by MEME (http://meme.nbcr.net/meme) to generate a consensus motif. FIMO software in the MEME suite was used to find similar sequences around the *agl1* gene. Significant sequences (*P*<0.003, calculated by FIMO) are listed in Table S4. 

## Results

### Atf1 and Pcr1 contribute to maltose utilization

**Figure 1:**
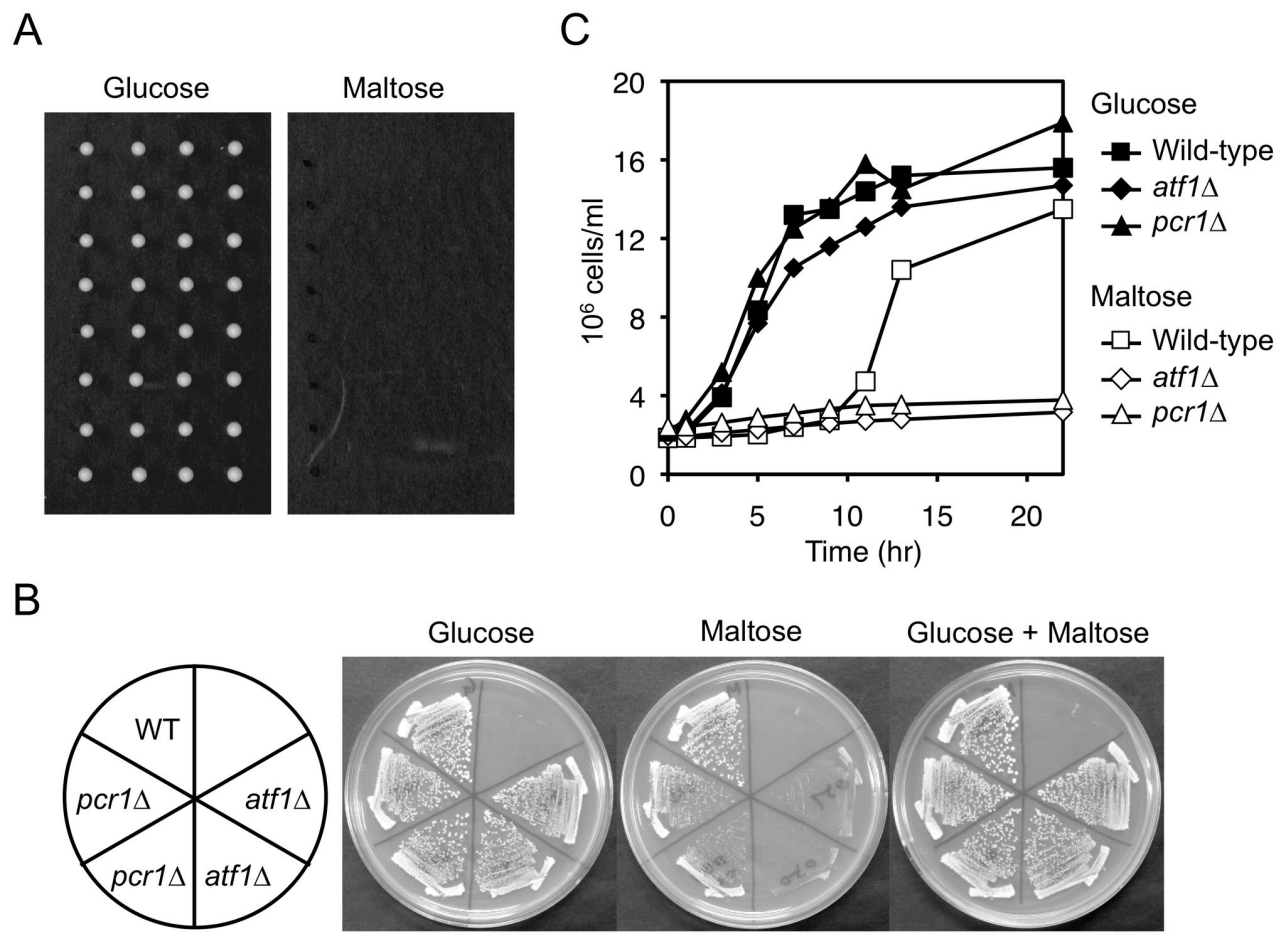
Atf1 and Pcr1 are required for efficient maltose utilization. (A) Fission yeast spores fail to form colonies when grown on maltose-containing medium. Wild-type (mating-type: *h* ^90^) tetrads were dissected on YE (glucose-containing) and YEM (maltose-containing) plates, and incubated at 30°C for 3 days. (B) *atf1∆* and *pcr1∆* mutants grow slowly on maltose-containing plates. Wild-type, *atf1∆*, and *pcr1∆* strains were streaked on plates containing the indicated sugar(s) and incubated at 30°C for 3 days. (C) Growth of *atf1∆* and *pcr1∆* mutants does not recover following switching from glucose- to maltose-containing medium. Cells of the indicated strains exponentially growing in YE were washed twice, resuspended in YE or YEM, and incubated at 30°C for the indicated number of hours.

In a routine genetic analysis of fission yeast, we noticed that spores did not germinate on plates that contained 3% maltose instead of 3% glucose (Figure 1A), indicating that spores cannot utilize maltose. We identified *atf1* and *pcr1* in a reverse genetic screen of genes that contribute to maltose hydrolysis. Although *atf1∆* and *pcr1∆* cells grow slightly slower than wild-type cells on YE plates that contain glucose as a carbon source [11,32], *atf1∆* cells grew markedly slower than wild-type cells on YEM plates (Figure 1B). *pcr1∆* cells also grew slower than wild-type cells on YEM plates, but only when the *pcr1∆* and wild-type cells were well-separated from each other (Figure 1B). *atf1∆* and *pcr1∆* cells did not show a severe growth defect when grown on a plate containing both maltose and glucose (Figure 1B), indicating that maltose is not toxic to these mutants and that Atf1 and Pcr1 are required for efficient utilization of maltose. Wild-type cells exponentially growing in YE medium at 30°C temporarily stopped growing when the carbon source was changed from glucose to maltose (Figure 1C). About 10 hours after this switch, the cells resumed growing. The timing of this growth recovery was dependent on the density of cells when the carbon source was switched (data not shown), suggesting that growth resumes when the cells have secreted a sufficient amount of maltase. *atf1∆* and *pcr1∆* mutants also stopped growing when the carbon source was changed; however, unlike wild-type cells, they did not resume growing (Figure 1C). These data indicate that Atf1 and Pcr1 are required for efficient maltose utilization.

### The extracellular maltase Agl1 is required for efficient utilization of maltose

**Figure 2:**
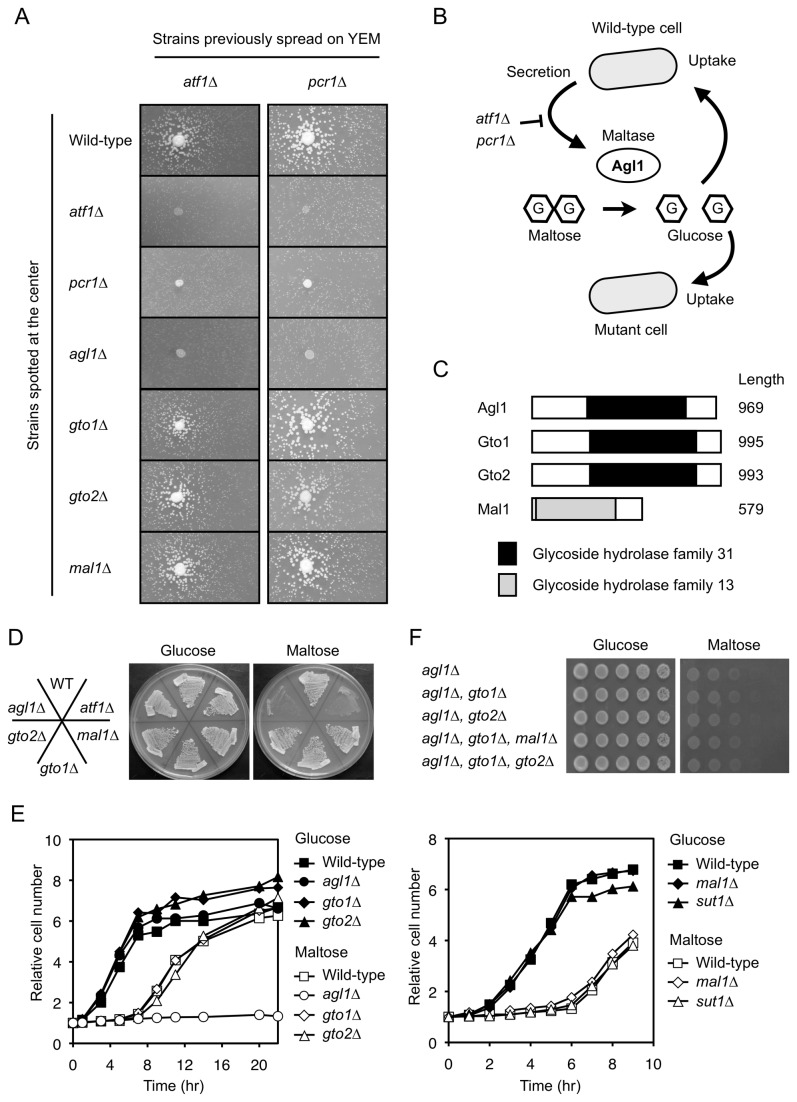
Agl1 is responsible for efficient maltose utilization. (A) Suppression of the slow growth of *atf1∆* and *pcr1∆* mutants on maltose-containing medium by neighboring colonies requires the *agl1* gene. Strains (indicated on the left) were spotted onto the centers of YEM plates on which *atf1∆* or *pcr1∆* cells had been spread, and were incubated for 3 days at 30°C. (B) Model describing how neighboring wild-type colonies suppress the slow growth of mutant cells. Wild-type cells secrete the extracellular maltase Agl1 in an Atf1- and Pcr1-dependent manner. Agl1 hydrolyzes maltose into glucose, which is then taken up and utilized by the neighboring mutant cells. (C) Schematic representation of the four possible maltases in fission yeast. The positions of the glycoside hydrolase domains and the lengths (number of amino acids) of each protein are indicated. (D) The *agl1∆* mutant grows slowly on maltose-containing plates. The indicated strains were streaked onto plates containing the indicated sugar and incubated at 30°C for 3 days. (E) Growth of the *agl1∆* mutant does not recover following switching from glucose- to maltose- containing medium. Cells of the indicated strains exponentially growing in YE were washed twice, resuspended in YE or YEM, and incubated at 30°C for the indicated number of hours. (F) Additional deletion of possible maltase-encoding genes does not enhance the slow growth phenotype of *agl1∆* cells on YEM plates. The indicated strains were serially diluted, spotted onto plates containing the indicated sugars, and incubated at 30°C for 5 days. Note: wild-type cells must not be spotted onto the same plates in this experiment because they secrete Agl1 and thereby affect the growth of neighboring mutant colonies.

On YEM plates, *atf1∆* and *pcr1∆* mutant colonies positioned close to wild-type colonies appeared to grow faster than those positioned further away (Figure 1B, data not shown). To confirm this phenomenon, wild-type or mutant cells were spotted onto the centers of YEM plates on which *atf1∆* or *pcr1∆* cells had been spread (Figure 2A). Mutant cells spread on YEM plates grew slowly and formed small colonies (Figure 2A). *atf1∆* and *pcr1∆* mutant colonies spotted onto the centers of plates did not affect the growth of surrounding mutant colonies that had been previously spread (Figure 2A). However, mutant colonies positioned close to central wild-type colonies grew faster than those positioned further away (Figure 2A). This suggested that maltase secreted by the wild-type cells hydrolyzed maltose into glucose, which was then taken up by the mutant cells (Figure 2B). Fission yeast has four genes encoding possible maltases (alpha-glucosidase) (Figure 2C). The *agl1* gene encodes the secreted maltase Agl1 [3,4], which belongs to the glycoside hydrolase family 31. The *SPAC1039.11c* and *SPAC30D11.01c* genes (hereafter denoted as *gto1* and *gto2*, respectively, for glycoside hydrolase family *t*hirty *o*ne) encode uncharacterized proteins that belong to the same family as Agl1. Mal1, encoded by *mal1*, belongs to the glycoside hydrolase family 13 and exhibits maltase activity *in vitro* [6]. Of the four maltase candidate genes, only *agl1* was clearly required for normal growth on YEM plates (Figure 2D). When *agl1*∆ cells were spotted onto the centers of plates, the growth rates of surrounding *atf1∆* and *pcr1∆* mutant colonies did not increase (Figure 2A). By contrast, when *gto1∆*, *gto2∆*, or *mal1∆* mutants were spotted onto the centers of plates, the growth rates of surrounding *atf1∆* and *pcr1∆* mutant colonies increased (Figure 2A). In agreement with these observations, growth of the *agl1∆* mutant did not recover after the carbon source was changed from glucose to maltose (Figure 2E), whereas growth of the other three mutants did recover (Figure 2E). In addition, deletion of the *sut1* gene, which encodes a maltose transporter [5], was not required for growth recovery following the carbon source change (Figure 2E). Furthermore, the slow growth phenotype of the *agl1∆* mutant observed on YEM plates was not enhanced by additional deletion of the other maltase candidate genes (Figure 2F). The slow growth phenotype is caused by the lack of Agl1 secretion because it was rescued by neighboring wild-type colonies but not by *agl1∆* mutant colonies (Figure S1). These data indicate that, of the four maltase candidates, Agl1 is predominantly responsible for efficient maltose utilization in fission yeast, that Agl1 secreted from neighboring wild-type colonies contributes to the faster growth of *atf1∆* and *pcr1∆* mutant colonies, and that Atf1 and Pcr1 are required for expression of Agl1. 

### Sty1 is not required for efficient utilization of maltose

As Atf1 is known to be a target of the Sty1 MAPK [17], it is possible that Sty1 also contributes to efficient maltose utilization. In order to examine whether Sty1 plays an important role in maltose utilization, we cultured *sty1∆* cells on YE, YEM and YE plates containing high concentration of KCl. *sty1∆* cells failed to grow on YE plates containing KCl as previously reported [17,33] (Figure S2A). However, *sty1∆* cells did not show any growth defect on YEM plates (Figure S2A). In agreement with this observation, neighboring *sty1∆* colonies successfully rescued the growth defect of *agl1∆* colonies (Figure S2B). These data indicate that Sty1 is dispensable for maltose secretion and hence for efficient maltose utilization.

### Atf1- and Pcr1-dependent transcriptional induction of the secreted maltase Agl1

**Figure 3:**
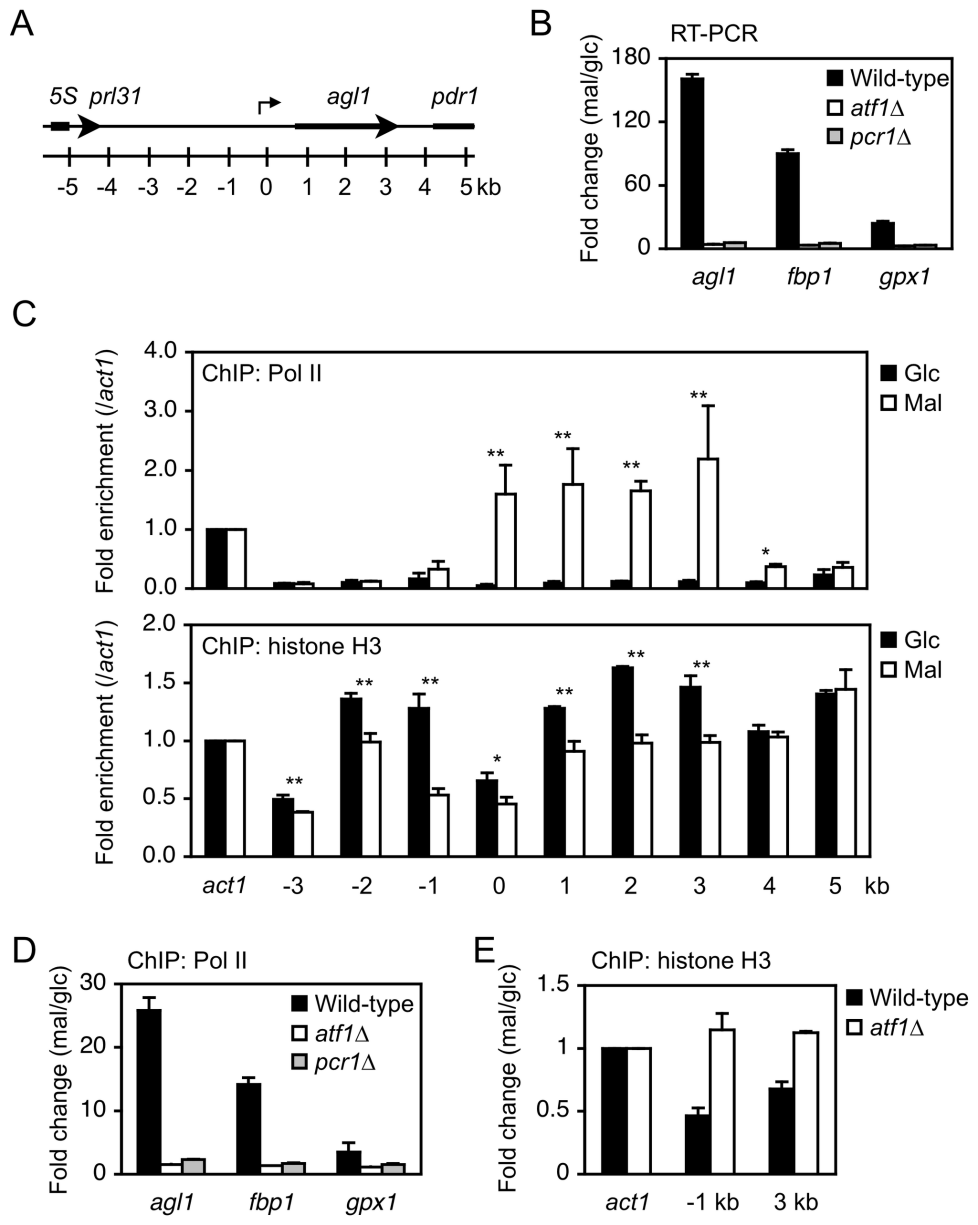
Atf1 and Pcr1 positively regulate the expression of Agl1 at the transcriptional level. (A) Schematic representation of the *agl1* locus. Positions relative to the transcriptional start site of *agl1* are indicated. (B) Atf1 and Pcr1 are required to increase the mRNA level of *agl1*. Cells of the indicated strains exponentially growing in YE were washed twice, resuspended in YE or YEM, and incubated at 30°C for 2 hours. Total RNA was used for RT-PCR analysis. Expression levels of the indicated genes relative to the level of the constitutive *act1* gene in the indicated strains grown in maltose-containing medium. The fold change compared to levels in cells grown in glucose-containing medium is shown. (C) RNA polymerase II and histone H3 occupancy of *agl1* before and after the carbon source change. Wild-type cells cultured as described in (B) were used for ChIP analysis. The levels of RNA polymerase II and histone H3 at target regions of *agl1* that are presented in (A) were normalized against their levels at *act1*. Glc: glucose; Mal: maltose; Pol II: RNA polymerase II. *P<0.05, **P<0.01 (Student's t-test). (D) The increased RNA polymerase II occupancy at *agl1* depends on Atf1 and Pcr1. Cells of the indicated strains were cultured as described in (B) and then used for ChIP analysis of RNA polymerase II. Levels of RNA polymerase II associated with the indicated genes relative to the level associated with *act1* in cells grown in maltose-containing medium. The fold change compared to levels in cells grown in glucose-containing medium is shown. (E) The decreased histone H3 occupancy at *agl1* depends on Atf1. Cells of the indicated strains were cultured as described in (B) and then used for ChIP analysis of histone H3 as described in (D). Error bars, s.d. (n=3).

The aforementioned results suggest that the transcription factors Atf1 and Pcr1 regulate Agl1 at the transcriptional level. The *agl1* gene has a long upstream intergenic region (Figure 3A). We performed qRT-PCR to determine whether this gene is regulated at the transcriptional level. In wild-type cells, the level of *agl1* mRNA drastically increased when the carbon source was changed from glucose to maltose (Figure 3B). By contrast, this was not observed in *atf1∆* or *pcr1∆* mutant cells (Figure 3B), indicating that the increase in *agl1* mRNA is dependent on Atf1 and Pcr1. To verify that this increase occurred at the transcriptional level, we performed ChIP analyses of RNA polymerase II using antibodies against the C-terminal heptad repeat of Rpb1 and the C-terminal domain of histone H3. After the carbon source was changed from glucose to maltose, RNA polymerase II occupancy at *agl1* increased (0–3 kb in Figure 3C, upper panel). Accordingly, the enrichment level of histone H3 decreased, both at the promoter and coding region (-3–3 kb in Figure 3C, lower panel). This resembles the nucleosome disassembly that occurs at activated genes [16,34,35]. Under these conditions, the *fbp1* and *gpx1* genes, which encode a fructose-1,6-bisphosphatase and a glutathione peroxididase, respectively, and are induced upon various stresses in an Atf1-dependent manner [13,16,19,36], were also upregulated at the transcriptional level (Figure 3B, D). On the other hand, neither transcriptional induction nor increased RNA polymerase II occupancy at these trans-activated loci were observed in *atf1∆* or *pcr1∆* mutant cells (Figure 3B, D). Consistent with this, the levels of histone H3 at the promoter and coding region of *agl1* were not decreased in *atf1∆* cells (Figure 3E). These results strongly suggest that Atf1 and Pcr1 positively regulate the transcription of *agl1*, as well as *fbp1* and *gpx1*, in response to switching of the carbon source from glucose to maltose. 

### Atf1 and Pcr1 associate with target genes

**Figure 4:**
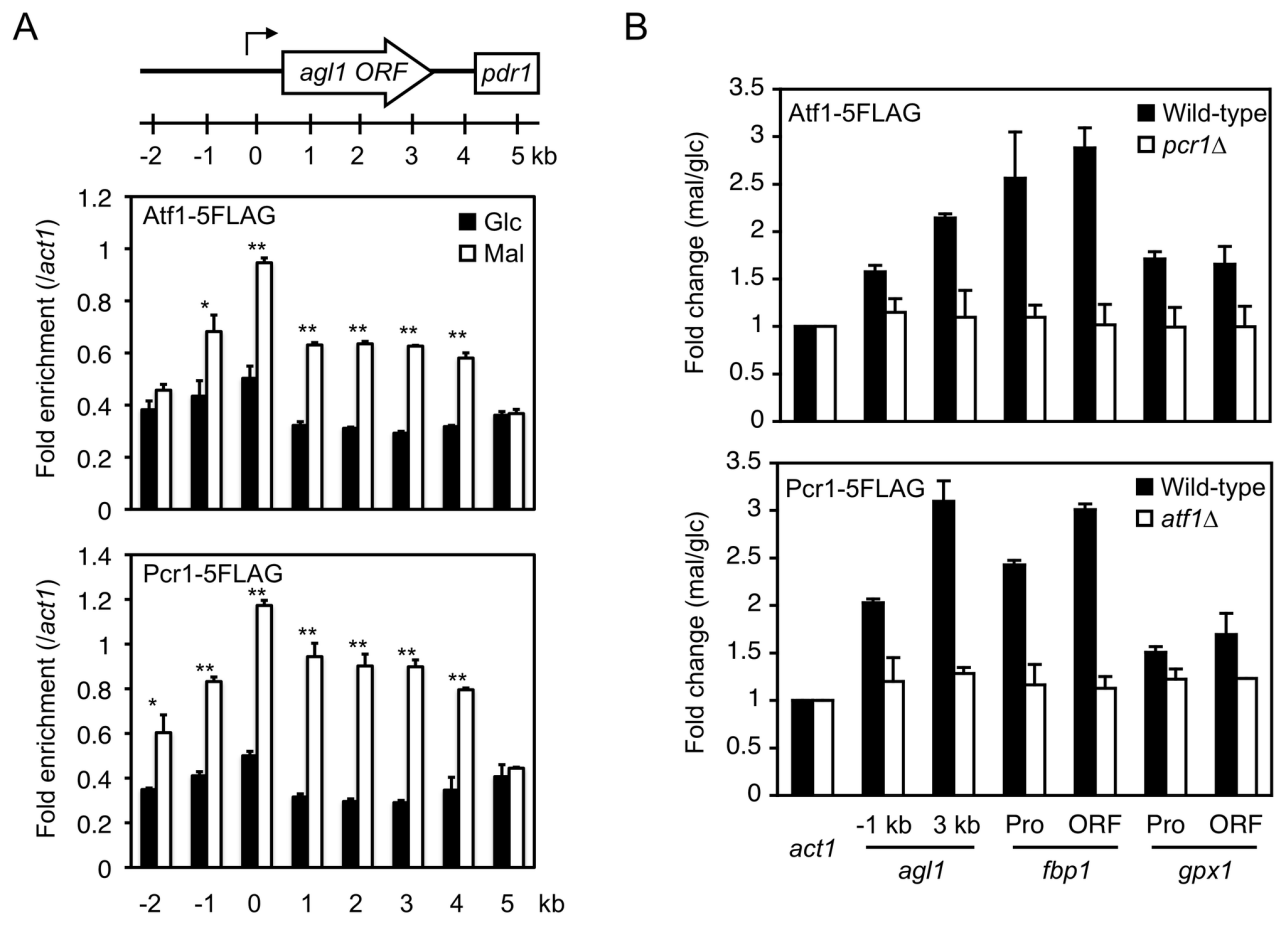
Atf1 and Pcr1 associate with their target genes upon switching of the carbon source from glucose to maltose in a manner dependent on each other. (A) Atf1 and Pcr1 associate with *agl1* in maltose-utilizing cells. Cells expressing the indicated proteins were cultured as described in Figure 3B and used for ChIP analysis. The levels of the indicated proteins at the target regions of *agl1* (presented in Figure 3A) were normalized against their levels at *act1*. Glc: glucose; Mal: maltose. *P<0.05, **P<0.01 (Student's t-test). (B) The increased level of Atf1 at *agl1* depends upon Pcr1, and vice versa. Cells of the indicated strains were cultured as described in Figure 3B and used for ChIP analysis. The levels of the indicated proteins associated with the indicated genes relative to the levels associated with *act1* in cells grown in maltose-containing medium. The fold change compared to levels in cells grown in glucose-containing medium is shown. Error bars, s.d. (n=3).

The *in vivo* binding sites of Atf1 and Pcr1 are degenerate in sequence and do not always match the TGACGT motif [14]. By surveying the degenerate sequences, potential binding sites of Atf1 and Pcr1 were identified interspersed in the promoter and coding region of *agl1* (Table S4). Therefore, we next analyzed the distribution patterns of Atf1 and Pcr1 around the *agl1* gene before and after the carbon source was changed. Enrichment of Atf1 and Pcr1 at the *pdr1* gene, which is downstream of *agl1*, did not change after the carbon source was changed (5 kb in Figure 4A). By contrast, Atf1 and Pcr1 associated preferentially with the promoter, and to a lesser extent with the coding region, of *agl1* after the carbon source was changed from glucose to maltose (Figure 4A). The associations of Atf1 and Pcr1 with the coding regions of *fbp1* and *gpx1* also increased when the carbon source was changed (Figure 4B), suggesting that Atf1 and Pcr1 associate with the coding regions of their target genes. The increased enrichment of Atf1 at its target genes was abolished when Pcr1 was depleted, and vice versa (Figure 4B), indicating that these two transcription factors act as a heteromer. These observations also strongly suggest that Atf1-Pcr1 heteromers associate with the promoters and coding regions of target genes, including *agl1*, in response to environmental changes. 

### Med7 interacts with Atf1-Pcr1 target genes

**Figure 5:**
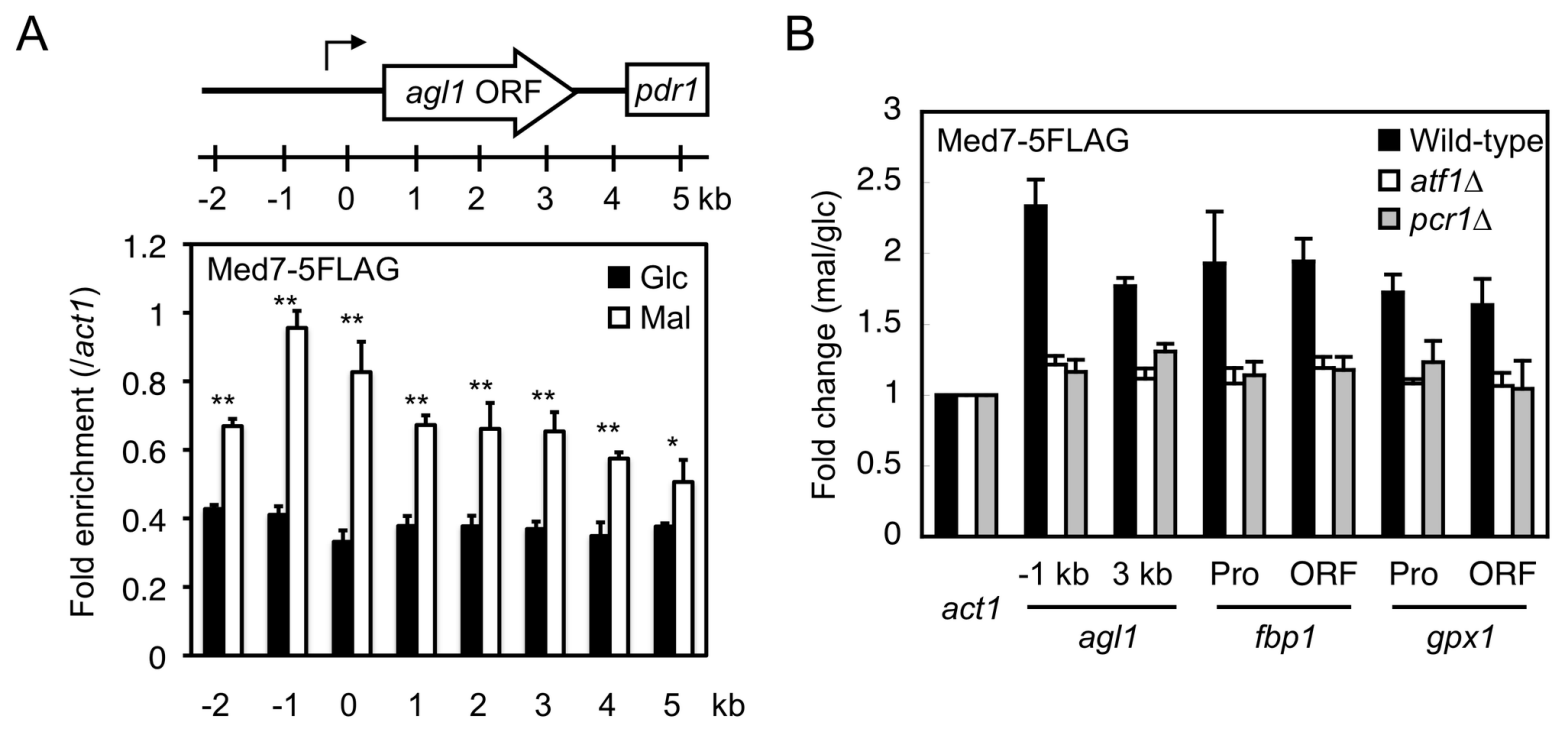
Med7 associates with its target genes upon switching of the carbon source from glucose to maltose in an Atf1- and Pcr1-dependent manner. (A) Med7 associates with *agl1* in maltose-utilizing cells. Cells expressing the indicated proteins were cultured as described in Figure 3B and used for ChIP analysis. The levels of the indicated proteins at the target regions of *agl1* (presented in Figure 3A) were normalized against the levels at *act1*. Glc: glucose; Mal: maltose. *P<0.05, **P<0.01 (Student's t-test). (B) The increased Med7 occupancy at *agl1* depends on Atf1 and Pcr1. Cells of the indicated strains were cultured as described in Figure 3B and used for ChIP analysis. The levels of Med7 associated with the indicated genes relative to the level of Med7 associated with *act1* in cells grown in maltose-containing medium. The fold change compared to levels in cells grown in glucose-containing medium is shown. Error bars, s.d. (n=3).

The dependence of Atf1 on Pcr1 for its association with target genes, and vice versa, suggests that the Atf1-Pcr1 heteromer has an important role in transcriptional activation. The recruitment of Mediator to target genes is dependent on transcription factors [23]. If Atf1 and Pcr1 contribute to the recruitment of Mediator to target genes, Med7, an essential subunit of Mediator, should interact with activated target genes in an Atf1- and Pcr1-dependent manner. This was indeed the case. Enrichment of Med7 at the promoter of *agl1* (-1 and 0 kb) increased when the carbon source was changed from glucose to maltose (Figure 5A). Med7 was also enriched at the coding region (1–3 kb), although to a lesser extent than at the promoter, implying that Mediator also has a function following transcriptional initiation, as proposed previously [37,38]. Med7 similarly associated with the coding regions of *fbp1* and *gpx1* (Figure 5B). Moreover, the association of Med7 with the target genes was dependent on Atf1 and Pcr1 (Figure 5B). These results indicate that Atf1 and Pcr1 are required for the proper localization of Mediator to the target gene.

## Discussion

We demonstrated that the extracellular maltase Agl1 is required for efficient utilization of maltose by the fission yeast *S. pombe*. Agl1 expression was induced at the transcriptional level in response to switching of the carbon source from glucose to maltose. The transcriptional induction of *agl1* required Atf1 and Pcr1, which associated with *agl1* after the carbon source was changed. These transcription factors predominantly associated with the transcriptional start sites of their target genes, and to a lesser extent with the coding regions. In agreement with these observations, *agl1* has previously been annotated as a potential target of Atf1 and Pcr1 [14]. 

 The degenerate TGACGT motifs interspersed around the *agl1* locus may assist the association of Atf1 and Pcr1 with chromatin. Degenerate hexamer sequences can be found throughout the genome. For instance, a hexamer sequence with one exchangeable nucleotide can exist in a DNA duplex of about 0.5 kb if the GC content is 50%. Therefore, the Atf1-Pcr1 binding pattern should be controlled by the chromatin structure, rather than simply by the presence of TGACGT motifs. Indeed, Atf1 and Pcr1 preferentially associate with promoter regions [14] that are not filled with nucleosomes [39]. This is likely because such regions are particularly accessible for Atf1 and Pcr1 [40]. If the accessibility of chromatin is the major determinant controlling Atf1-Pcr1 binding, these transcription factors should bind “open” promoters (i.e., not filled with nucleosomes) prior to exposure to environmental stress and their binding may not change in response to this stress. This model fits with the observation that Atf1 and Pcr1 constitutively bind the majority of the promoters of stress-responsive genes [14]. 

 The Atf1-dependent reduction in the level of histone H3 around the *agl1* locus suggests that nucleosomes that might have masked potential Atf1-Pcr1 binding sites have been removed, allowing Atf1 and Pcr1 to bind. The enrichment of Atf1 and Pcr1 increased predominantly at the transcriptional start site. This suggests that these transcription factors directly contribute to the induction of *agl1* transcription. The lower enrichment of Atf1 and Pcr1 at the coding region of *agl1* suggests that this association occurs due to nucleosomes being removed during transcription. Alternatively, the binding of Atf1 and Pcr1 to the coding region could maintain *agl1* in an open state necessary for transcriptional elongation. The well-known Atf1-target genes, *fbp1* and *gpx1*, were also induced when the carbon source was switched from glucose to maltose. Accordingly, Atf1 and Pcr1 associated with the promoters and coding regions of these genes. Therefore, association with coding regions may be a general characteristic of these transcription factors. 

 The association of Atf1 and Pcr1 with coding regions of their target genes has not been reported previously. This is probably because the levels of Atf1 and Pcr1 are lower at these regions than at promoters. It is possible that this association of Atf1 and Pcr1 facilitates the previously reported association of Sty1 with coding regions of induced genes that occurs in an Atf1- and Pcr1-dependent manner [12]. Interestingly, Sty1 turned out to be dispensable for maltase secretion and hence for normal growth on maltose medium, suggesting that Sty1 is not (or less) involved in the transcriptional induction of the *agl1* gene. Therefore, the *agl1* gene is not suitable for examining the involvement of Atf1 and Pcr1 in Sty1 association with coding regions. The Mediator subunit Med7 associated with the promoters and coding regions of induced genes (*agl1*, *fbp1*, and *gpx1*), suggesting that Mediator plays an important role in the transcriptional induction of these genes. Med7 was predominantly enriched at the -1 kb region of *agl1* and was not located at the position where Atf1 and Pcr1 were mostly enriched (0 kb). Therefore, although the recruitment of Mediator to Atf1- and Pcr1-target genes is dependent on these transcription factors, other factors might determine the localization pattern of Mediator. 

 Here, we showed that the previously described maltose transporter Sut1 [5] and the previously described intracellular maltase Mal1 [6] do not appear to contribute to efficient maltose utilization in fission yeast. This observation supports the theory that fission yeast hydrolyze maltose outside the cell and uptake the generated glucose, and do not uptake maltose and then hydrolyze it inside cells [2]. Our data also indicate that the glycoside hydrolase family 31 proteins Gto1 and Gto2 are not required for efficient maltose utilization in fission yeast. The precise functions of these proteins in a physiological context should be clarified in future studies. 

 It is not known why fission yeast spores failed to germinate on plates containing maltose instead of glucose. It is possible that spores cannot detect maltose, and that transcriptional induction of *agl1* and/or the functions of factors that are required for secretion of Agl1 are repressed in spores. It also remains unknown whether the presence of maltose is sensed, how transcriptional induction of *agl1* by Atf1 and Pcr1 is regulated, or how Agl1 is secreted. These issues should also be addressed in the future. 

 Rhind N. et al. recently reported that the *agl1* gene, among others, was induced by glucose depletion [41]. They showed that fragments per kilobase per million reads (FPKM) value in RNA-seq experiments for the *agl1* gene was 21 in log-phase culture and it increased to 3,199 (152-fold induction) when glucose was depleted. The extent of induction observed when glucose was depleted is similar to that observed in our study (approximately 150-fold, see Figure 3B). Therefore, expression of the *agl1* gene appears to be induced simply by limiting glucose regardless of the presence of maltose.

 Our data showed that RNA polymerase II occupancy is moderately higher at *agl1* than at *act1*, which is also highly expressed. Consistently, in the RNA-seq analysis performed by Rhind et al. [41], FPKM values of the *agl1* gene in the glucose depleted culture is also moderately higher than that of *act1*. Therefore, the *agl1* promoter may be useful for massive production of recombinant proteins in cells. As Agl1 is a secreted protein, perhaps recombinant proteins could be secreted if fused to the uncharacterized secretory signal sequence of Agl1. Deletion of *agl1* did not cause acute cell death in maltose-containing medium. This suggests that if *agl1* was replaced with a gene of interest, the protein of interest could be efficiently and continuously generated. Therefore, the mechanisms that regulate Agl1 should be studied further to facilitate the efficient production of extracellular and intracellular recombinant proteins.

## Supporting Information

Figure S1 (TIFF)

Figure S2 (TIFF)

Table S1 **Oligonucleotide sequences used to disrupt and tag chromosomal genes.**
(XLS)

Table S2 **Oligonucleotide sequences used for qRT-PCR.**
(XLS)

Table S3 **Oligonucleotide sequences used for ChIP analysis.**
(XLS)

Table S4 **Degenerate **TGACGT** motifs around the *agl1* gene.**
(XLS)

## References

[1] Horák J (2013) Regulations of sugar transporters: insights from yeast. Curr Genet 59: 1-31. doi:10.1007/s00294-013-0388-8. PubMed: 23455612. 23455612

[2] Mehta SV, Patil VB, Velmurugan S, Lobo Z, Maitra PK (1998) std1, a gene involved in glucose transport in Schizosaccharomyces pombe. J Bacteriol 180: 674-679. 9457874 10.1128/jb.180.3.674-679.1998 PMC106938

[3] Okuyama M, Okuno A, Shimizu N, Mori H, Kimura A et al. (2001) Carboxyl group of residue Asp647 as possible proton donor in catalytic reaction of alpha-glucosidase from *Schizosaccharomyces* *pombe* . Eur J Biochem 268: 2270-2280. doi:10.1046/j.1432-1327.2001.02104.x. PubMed: 11298744. 11298744

[4] Jansen ML, Krook DJ, De Graaf K, van Dijken JP, Pronk JT et al. (2006) Physiological characterization and fed-batch production of an extracellular maltase of *Schizosaccharomyces* *pombe* CBS 356. FEMS Yeast Res 6: 888-901. doi:10.1111/j.1567-1364.2006.00091.x. PubMed: 16911511. 16911511

[5] Reinders A, Ward JM (2001) Functional characterization of the alpha-glucoside transporter Sut1p from *Schizosaccharomyces* *pombe*, the first fungal homologue of plant sucrose transporters. Mol Microbiol 39: 445-454. doi:10.1046/j.1365-2958.2001.02237.x. PubMed: 11136464. 11136464

[6] Chi Z, Ni X, Yao S (2008) Cloning and overexpression of a maltase gene from *Schizosaccharomyces* *pombe* in *Escherichia* *coli* and characterization of the recombinant maltase. Mycol Res 112: 983-989. doi:10.1016/j.mycres.2008.01.024. PubMed: 18556189. 18556189

[7] Steiner WW, Smith GR (2005) Optimizing the nucleotide sequence of a meiotic recombination hotspot in *Schizosaccharomyces* *pombe* . Genetics 169: 1973-1983. doi:10.1534/genetics.104.039230. PubMed: 15716492. 15716492 PMC1449614

[8] Wahls WP, Smith GR (1994) A heteromeric protein that binds to a meiotic homologous recombination hot spot: correlation of binding and hot spot activity. Genes Dev 8: 1693-1702. doi:10.1101/gad.8.14.1693. PubMed: 7958849. 7958849

[9] Jia S, Noma K, Grewal SI (2004) RNAi-independent heterochromatin nucleation by the stress-activated ATF/CREB family proteins. Science 304: 1971-1976. doi:10.1126/science.1099035. PubMed: 15218150. 15218150

[10] Shiozaki K, Russell P (1996) Conjugation, meiosis, and the osmotic stress response are regulated by Spc1 kinase through Atf1 transcription factor in fission yeast. Genes Dev 10: 2276-2288. doi:10.1101/gad.10.18.2276. PubMed: 8824587. 8824587

[11] Takeda T, Toda T, Kominami K, Kohnosu A, Yanagida M et al. (1995) *Schizosaccharomyces* *pombe* atf1^+^ encodes a transcription factor required for sexual development and entry into stationary phase. EMBO J 14: 6193-6208. PubMed: 8557039. 8557039 10.1002/j.1460-2075.1995.tb00310.x PMC394744

[12] Reiter W, Watt S, Dawson K, Lawrence CL, Bähler J et al. (2008) Fission yeast MAP kinase Sty1 is recruited to stress-induced genes. J Biol Chem 283: 9945-9956. doi:10.1074/jbc.M710428200. PubMed: 18252721. 18252721 PMC3668131

[13] Sansó M, Gogol M, Ayté J, Seidel C, Hidalgo E (2008) Transcription factors Pcr1 and Atf1 have distinct roles in stress- and Sty1-dependent gene regulation. Eukaryot Cell 7: 826-835. doi:10.1128/EC.00465-07. PubMed: 18375616. 18375616 PMC2394965

[14] Eshaghi M, Lee JH, Zhu L, Poon SY, Li J et al. (2010) Genomic binding profiling of the fission yeast stress-activated MAPK Sty1 and the bZIP transcriptional activator Atf1 in response to H_2_O_2_ . PLOS ONE 5: e11620. doi:10.1371/journal.pone.0011620. PubMed: 20661279. 20661279 PMC2905393

[15] Neely LA, Hoffman CS (2000) Protein kinase A and mitogen-activated protein kinase pathways antagonistically regulate fission yeast *fbp1* transcription by employing different modes of action at two upstream activation sites. Mol Cell Biol 20: 6426-6434. doi:10.1128/MCB.20.17.6426-6434.2000. PubMed: 10938120. 10938120 PMC86118

[16] Hirota K, Miyoshi T, Kugou K, Hoffman CS, Shibata T et al. (2008) Stepwise chromatin remodelling by a cascade of transcription initiation of non-coding RNAs. Nature 456: 130-134. doi:10.1038/nature07348. PubMed: 18820678. 18820678

[17] Wilkinson MG, Samuels M, Takeda T, Toone WM, Shieh JC et al. (1996) The Atf1 transcription factor is a target for the Sty1 stress-activated MAP kinase pathway in fission yeast. Genes Dev 10: 2289-2301. doi:10.1101/gad.10.18.2289. PubMed: 8824588. 8824588

[18] Takada H, Nishimura M, Asayama Y, Mannse Y, Ishiwata S et al. (2007) Atf1 is a target of the mitogen-activated protein kinase Pmk1 and regulates cell integrity in fission yeast. Mol Biol Cell 18: 4794-4802. doi:10.1091/mbc.E07-03-0282. PubMed: 17881729. 17881729 PMC2096581

[19] Lawrence CL, Maekawa H, Worthington JL, Reiter W, Wilkinson CR et al. (2007) Regulation of *Schizosaccharomyces* *pombe* Atf1 protein levels by Sty1-mediated phosphorylation and heterodimerization with Pcr1. J Biol Chem 282: 5160-5170. PubMed: 17182615. 17182615 10.1074/jbc.M608526200

[20] Holstege FC, Jennings EG, Wyrick JJ, Lee TI, Hengartner CJ et al. (1998) Dissecting the regulatory circuitry of a eukaryotic genome. Cell 95: 717-728. doi:10.1016/S0092-8674(00)81641-4. PubMed: 9845373. 9845373

[21] Björklund S, Gustafsson CM (2005) The yeast Mediator complex and its regulation. Trends Biochem Sci 30: 240-244. doi:10.1016/j.tibs.2005.03.008. PubMed: 15896741. 15896741

[22] Takagi Y, Kornberg RD (2006) Mediator as a general transcription factor. J Biol Chem 281: 80-89. doi:10.1074/jbc.M508253200. PubMed: 16263706. 16263706

[23] Casamassimi A, Napoli C (2007) Mediator complexes and eukaryotic transcription regulation: an overview. Biochimie 89: 1439-1446. doi:10.1016/j.biochi.2007.08.002. PubMed: 17870225. 17870225

[24] Krawchuk MD, Wahls WP (1999) High-efficiency gene targeting in *Schizosaccharomyces* *pombe* using a modular, PCR-based approach with long tracts of flanking homology. Yeast 15: 1419-1427. doi:10.1002/(SICI)1097-0061(19990930)15:13. PubMed: 10509024. 10509024 PMC3190138

[25] Bähler J, Wu JQ, Longtine MS, Shah NG, McKenzie A 3rd et al. (1998) Heterologous modules for efficient and versatile PCR-based gene targeting in *Schizosaccharomyces* *pombe* . Yeast 14: 943-951. doi:10.1002/(SICI)1097-0061(199807)14:10. PubMed: 9717240. 9717240

[26] Sato M, Dhut S, Toda T (2005) New drug-resistant cassettes for gene disruption and epitope tagging in *Schizosaccharomyces* *pombe* . Yeast 22: 583-591. doi:10.1002/yea.1233. PubMed: 15942936. 15942936

[27] Noguchi C, Garabedian MV, Malik M, Noguchi E (2008) A vector system for genomic FLAG epitope-tagging in *Schizosaccharomyces* *pombe* . Biotechnol J 3: 1280-1285. doi:10.1002/biot.200800140. PubMed: 18729046. 18729046

[28] Schmitt ME, Brown TA, Trumpower BL (1990) A rapid and simple method for preparation of RNA from *Saccharomyces* *cerevisiae* . Nucleic Acids Res 18: 3091-3092. doi:10.1093/nar/18.10.3091. PubMed: 2190191. 2190191 PMC330876

[29] Livak KJ, Schmittgen TD (2001) Analysis of relative gene expression data using real-time quantitative PCR and the 2(-Delta Delta C(T)) Method. Methods 25: 402-408. doi:10.1006/meth.2001.1262. PubMed: 11846609. 11846609

[30] Luu-The V, Paquet N, Calvo E, Cumps J (2005) Improved real-time RT-PCR method for high-throughput measurements using second derivative calculation and double correction. BioTechniques 38: 287-293. doi:10.2144/05382RR05. PubMed: 15727135. 15727135

[31] Kato H, Goto DB, Martienssen RA, Urano T, Furukawa K et al. (2005) RNA polymerase II is required for RNAi-dependent heterochromatin assembly. Science 309: 467-469. doi:10.1126/science.1114955. PubMed: 15947136. 15947136

[32] Watanabe Y, Yamamoto M (1996) *Schizosaccharomyces* *pombe* pcr1^+^ encodes a CREB/ATF protein involved in regulation of gene expression for sexual development. Mol Cell Biol 16: 704-711. PubMed: 8552099. 8552099 10.1128/mcb.16.2.704 PMC231050

[33] Millar JB, Buck V, Wilkinson MG (1995) Pyp1 and Pyp2 PTPases dephosphorylate an osmosensing MAP kinase controlling cell size at division in fission yeast. Genes Dev 9: 2117-2130. doi:10.1101/gad.9.17.2117. PubMed: 7657164. 7657164

[34] Kristjuhan A, Svejstrup JQ (2004) Evidence for distinct mechanisms facilitating transcript elongation through chromatin in vivo. EMBO J 23: 4243-4252. doi:10.1038/sj.emboj.7600433. PubMed: 15457216. 15457216 PMC524397

[35] Adkins MW, Williams SK, Linger J, Tyler JK (2007) Chromatin disassembly from the *PHO5* promoter is essential for the recruitment of the general transcription machinery and coactivators. Mol Cell Biol 27: 6372-6382. doi:10.1128/MCB.00981-07. PubMed: 17620413. 17620413 PMC2099613

[36] Madrid M, Soto T, Franco A, Paredes V, Vicente J et al. (2004) A cooperative role for Atf1 and Pap1 in the detoxification of the oxidative stress induced by glucose deprivation in *Schizosaccharomyces* *pombe* . J Biol Chem 279: 41594-41602. doi:10.1074/jbc.M405509200. PubMed: 15247218. 15247218

[37] Zhu X, Wirén M, Sinha I, Rasmussen NN, Linder T et al. (2006) Genome-wide occupancy profile of mediator and the Srb8-11 module reveals interactions with coding regions. Mol Cell 22: 169-178. doi:10.1016/j.molcel.2006.03.032. PubMed: 16630887. 16630887

[38] Andrau JC, van de Pasch L, Lijnzaad P, Bijma T, Koerkamp MG et al. (2006) Genome-wide location of the coactivator mediator: Binding without activation and transient Cdk8 interaction on DNA. Mol Cell 22: 179-192. doi:10.1016/j.molcel.2006.03.023. PubMed: 16630888. 16630888

[39] Lantermann AB, Straub T, Strålfors A, Yuan GC, Ekwall K et al. (2010) *Schizosaccharomyces* *pombe* genome-wide nucleosome mapping reveals positioning mechanisms distinct from those of *Saccharomyces* *cerevisiae* . Nat Struct Mol Biol 17: 251-257. doi:10.1038/nsmb.1741. PubMed: 20118936. 20118936

[40] Bell O, Tiwari VK, Thomä NH, Schübeler D (2011) Determinants and dynamics of genome accessibility. Nat Rev Genet 12: 554-564. doi:10.1038/nrg3017. PubMed: 21747402. 21747402

[41] Rhind N, Chen Z, Yassour M, Thompson DA, Haas BJ et al. (2011) Comparative functional genomics of the fission yeasts. Science 332: 930-936. doi:10.1126/science.1203357. PubMed: 21511999. 21511999 PMC3131103

