# Supplementary figures and images for: The Transcription Factors Atf1 and Pcr1 Are Essential for Transcriptional Induction of the Extracellular Maltase Agl1 in Fission Yeast
Source: PLoS One. 2013 Nov 5;8(11):e80572. doi: 10.1371/journal.pone.0080572 (PMC3818258; doi:10.1371/journal.pone.0080572)

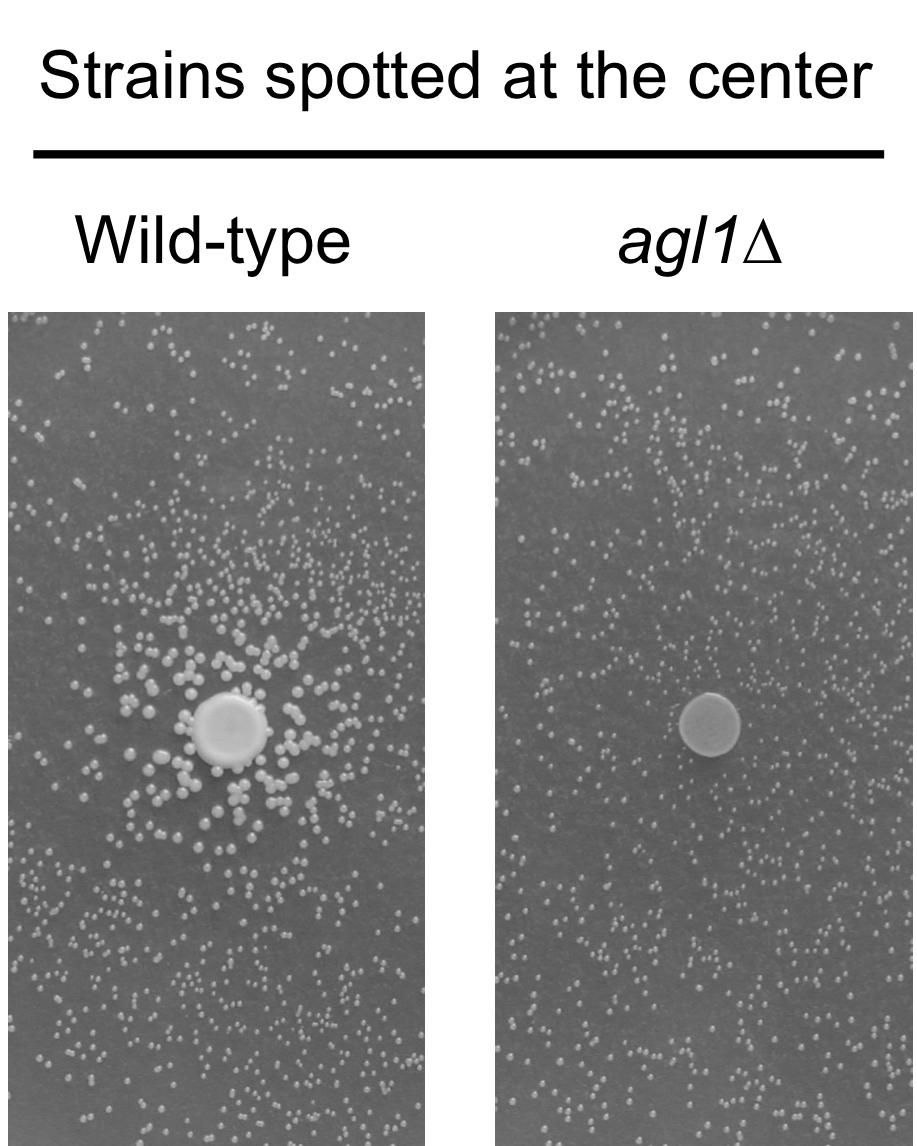

Supplement: Figure S1 — (TIFF) [file pone.0080572.s001.tiff]

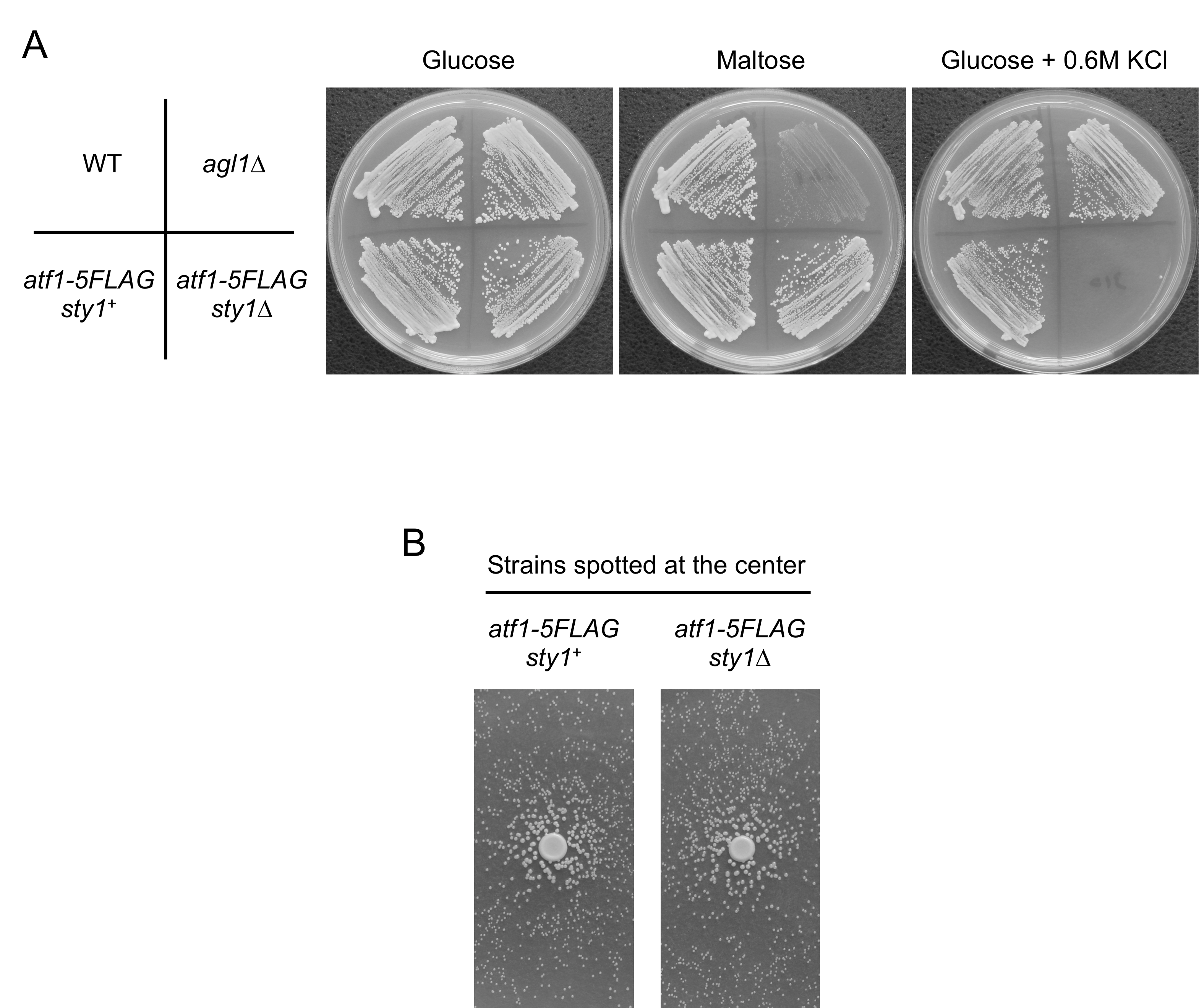

Supplement: Figure S2 — (TIFF) [file pone.0080572.s002.tiff]
